# Supplementary figures and images for: Formamide denaturation of double-stranded DNA for fluorescence in situ hybridization (FISH) distorts nanoscale chromatin structure
Source: PLoS One. 2024 May 28;19(5):e0301000. doi: 10.1371/journal.pone.0301000 (PMC11132451; doi:10.1371/journal.pone.0301000)

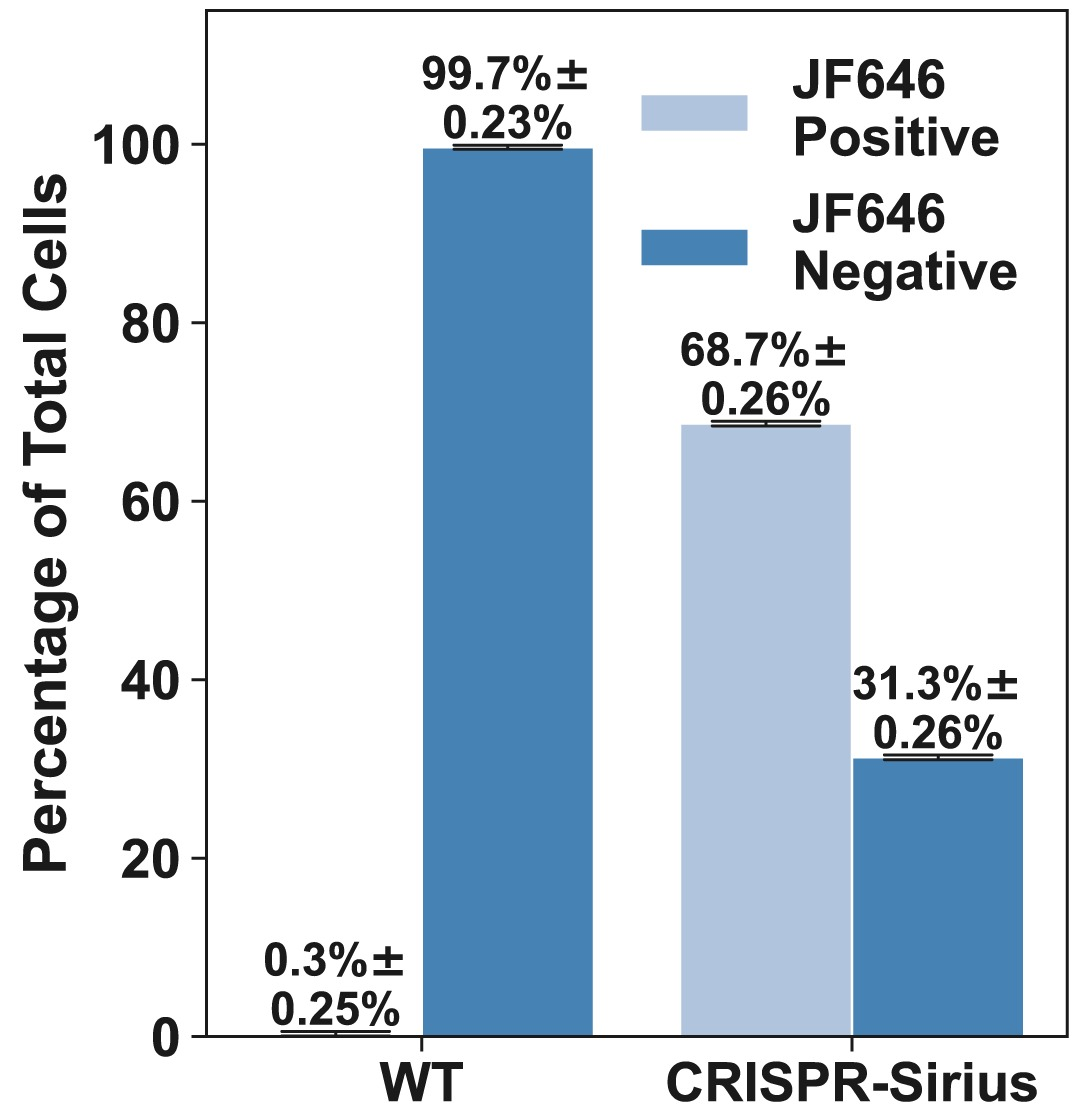

Supplement: S1 Fig — CRISPR-Sirius transduction efficiency as determined by the number of cells exhibiting fluorescence measured using flow cytometry. The XXYLT1 gene was labeled with the MS2 aptamer and stained using Janelia Fluorophore 646 for visualization. In the control sample, ~80,000 live cells were captured by flow cytometry, while the CRISPR-Sirius sample had ~70,000 live cells. (TIF) [file pone.0301000.s001.tif]

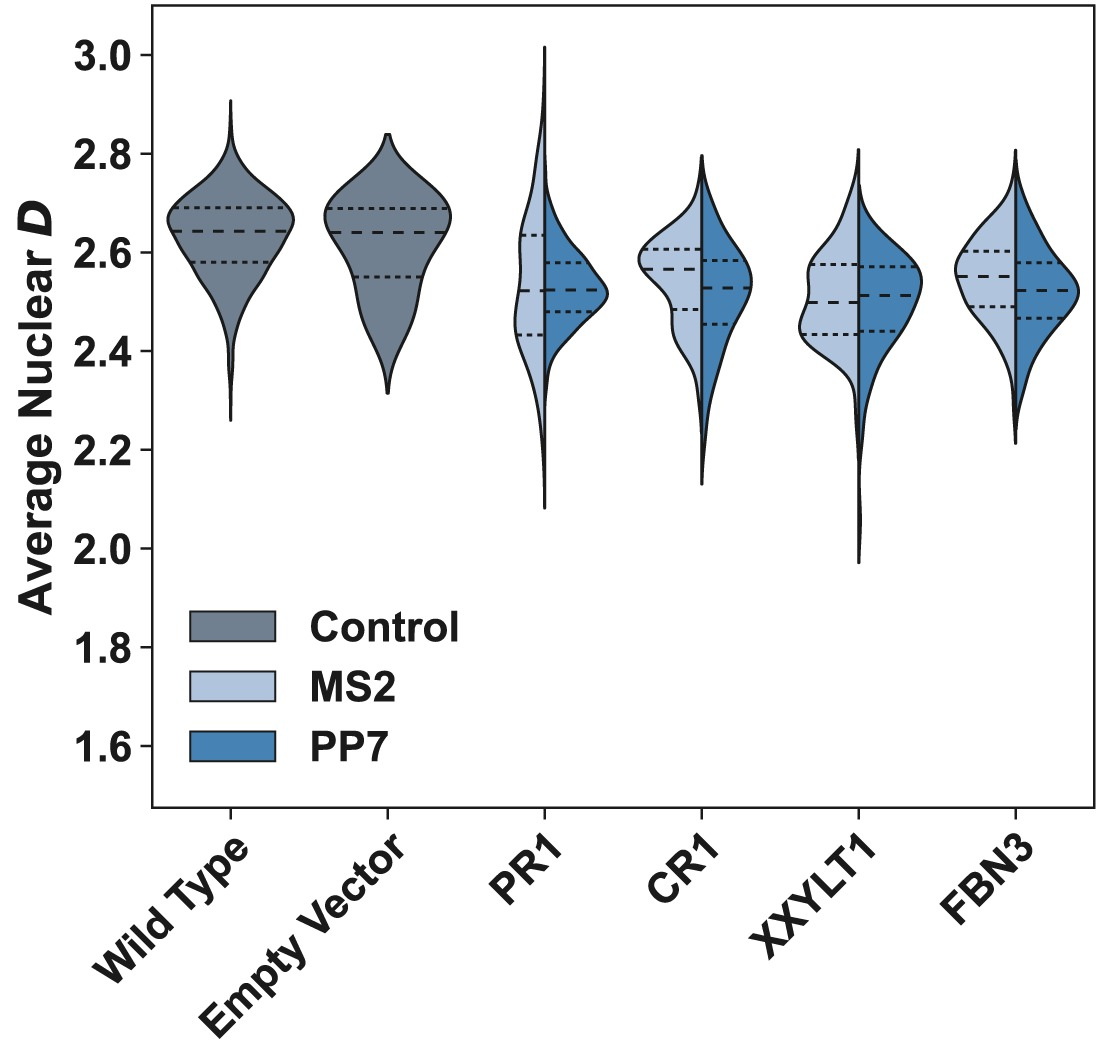

Supplement: S2 Fig — PWS microscopy imaging of cells labeled with CRISPR-Sirius using two different aptamers (MS2 and PP7) and four primers targeting the pericentromeric region (PR1) on chromosome 19, repeats on the gene which codes for complement receptor 1 (CR1) on chromosome 1, an intronic region on the gene for xyloside xylosyltransferase 1 (XXYLT1) on chromosome 3, and repeats on an intron for the gene encoding fibrillin 3 (FBN3) on chromosome 19. Average nuclear D decreases slightly for both aptamers and all primers, however, all CRISPR populations reach similar mean D values after lentiviral transduction, showing that the choice of guide RNA and aptamer is not the driving factor for chromatin changes. Violins include data from between 40–1100 nuclei. Dashed lines within violins denote the 75th percentile, median, and 25th percentile from top to bottom. (TIF) [file pone.0301000.s002.tif]
