# Supplementary material for: Formamide denaturation of double-stranded DNA for fluorescence in situ hybridization (FISH) distorts nanoscale chromatin structure
Source: PLoS One. 2024 May 28;19(5):e0301000. doi: 10.1371/journal.pone.0301000 (PMC11132451; doi:10.1371/journal.pone.0301000)
Supplement: S1 Table — (DOCX) [file pone.0301000.s003.docx]

| **Fixative** | **Average Nuclear *D*** ±  **Standard Deviation** | | **% Change from Live Cells** | | **% Change from Fixed Cells** |
| --- | --- | --- | --- | --- | --- |
|  | **Fixed Cells** | **Formamide Treated Cells** | **Fixed Cells** | **Formamide Treated Cells** |  |
| **Live Cells** | 2.63±0.09 (N=1039 nuclei) | | N/A | | N/A |
| **10 min**  **4% PFA** | 2.43±0.16 (N=144 nuclei) | 2.18±0.12 (N=147 nuclei) | -7.62%  (p=3.24x10^-88^) | -17.04%  (p=1.45x10^-313^) | -10.19% (p=1.22x10^-35^) |
| **30 min**  **4% PFA** | 2.64±0.06 (N=115 nuclei) | 2.28±0.15 (N=121 nuclei) | 0.24%  (p=1) | -13.33%  (p=2.54x10^-193^) | -13.53% (p=8.24x10^-61^) |
| **90 min**  **4% PFA** | 2.59±0.07 (N=129 nuclei) | 2.33±0.10 (N=130 nuclei) | -1.42%  (p=6.86x10^-4^) | -11.46%  (p=4.13x10^-182^) | -10.18% (p=4.49x10^-67^) |
| **10 min**  **2% PFA+ 2.5% GA** | 2.62±0.09 (N=131 nuclei) | 2.44±0.11 (N=143 nuclei) | -0.42%  (p=1) | -7.30% (p=1.72x10^-93^) | -6.91% (p=4.33x10^-34^) |
| **10 min**  **Ethanol** | 2.84±0.05 (N=122 nuclei) | 2.50±0.08 (N=118 nuclei) | 7.85%  (p=3.01x10^-105^) | -4.96%  (p=9.96x10^-44^) | -11.87% (p=2.28x10^-105^) |
| **10 min Methanol** | 2.62±0.07 (N=113 nuclei) | 2.21±0.12 (N=147 nuclei) | -0.43%  (p=1) | -16.11%  (p=2.34x10^-292^) | -15.75% (p=1.01x10^-89^) |
