# Supplementary material for: Formamide denaturation of double-stranded DNA for fluorescence in situ hybridization (FISH) distorts nanoscale chromatin structure
Source: PLoS One. 2024 May 28;19(5):e0301000. doi: 10.1371/journal.pone.0301000 (PMC11132451; doi:10.1371/journal.pone.0301000)
Supplement: S2 Table — (DOCX) [file pone.0301000.s004.docx]

| **Condition** | | **Average Nuclear *D*** ±  **Standard Deviation** | **% Change from Live Cells** | **% Change from Fixed Cells** |
| --- | --- | --- | --- | --- |
| **Live Cells** | | 2.58±0.09  (N=427 nuclei) | N/A | N/A |
| **Fixed Cells** | | 2.46±0.15  (N=419 nuclei) | -4.66%  (p=1.23x10^-40^) | N/A |
| **Heat Denaturation** | **70°C** | 2.06±0.15  (N=425 nuclei) | -20.05%  (p=7.44x10^-318^) | -16.15%  (p=1.56x10^-189^) |
|  | **75°C** | 2.11±0.16  (N=395 nuclei) | -18.10%  (p=9.27x10^-266^) | -14.10%  (p=1.29x10^-146^) |
|  | **80°C** | 2.16±0.24  (N=396 nuclei) | -16.37%  (p=9.22x10^-160^) | -12.29%  (p=9.66x10^-83^) |
| **Overnight Incubation** | **12-15 Hours** | 2.21±0.12  (N=430 nuclei) | -14.19%  (p=1.54x10^-252^) | -10.00%  (p=2.00x10^-109^) |
|  | **24 Hours** | 2.26±0.14  (N=332 nuclei) | -12.60%  (p=1.07x10^-181^) | -8.33%  (p=9.42x10^-66^) |
|  | **48 Hours** | 2.28±0.14  (N=216 nuclei) | -11.74%  (p=3.66x10^-143^) | -7.42%  (p=1.98x10^-42^) |
