# Supplementary material for: Formamide denaturation of double-stranded DNA for fluorescence in situ hybridization (FISH) distorts nanoscale chromatin structure
Source: PLoS One. 2024 May 28;19(5):e0301000. doi: 10.1371/journal.pone.0301000 (PMC11132451; doi:10.1371/journal.pone.0301000)
Supplement: S3 Table — (DOCX) [file pone.0301000.s005.docx]

| **sgRNA** | **Average Nuclear *D*** ±  **Standard Deviation** | | **% Change from Wild Type** | | **% Change from Empty Vector** | | **% Difference Between MS2 and PP7** |
| --- | --- | --- | --- | --- | --- | --- | --- |
|  | **MS2** | **PP7** | **MS2** | **PP7** | **MS2** | **PP7** |  |
| **Wild Type** | 2.63±0.08 (N=1081 nuclei) | | N/A | | N/A | | N/A |
| **Empty Vector** | 2.62±0.10 (N=41 nuclei) | | -0.60% (p=1) | | N/A | | N/A |
| **PR1** | 2.54±0.14  (N=313  nuclei) | 2.53±0.07  (N=798  nuclei) | -3.68%  (p=2.92x  10^-46^) | -3.93%  (p=2.26x  10^-141^) | -3.09%  (p=0.021) | -3.34%  (p=3.18x  10^-11^) | 0.26%  (p=1) |
| **CR1** | 2.54±0.09  (N=88  nuclei) | 2.51±0.11  (N=89  nuclei) | -3.34%  (p=7.26x  10^-19^) | -4.64%  (p=2.61x  10^-34^) | -2.76%  (p=0.002) | -4.06%  (p=9.12x  10^-6^) | 1.35%  (p=0.898) |
| **XXYLT1** | 2.51±0.09  (N=80  nuclei) | 2.49±0.10  (N=80  nuclei) | -4.67%  (p=1.98x  10^-32^) | -5.26%  (p=7.83x  10^-40^) | -4.09%  (p=1.31x  10^-6^) | -4.69%  (p=1.89x  10^-7^) | 0.63%  (p=1) |
| **FBN3** | 2.55±0.08  (N=84  nuclei) | 2.52±0.09  (N=86  nuclei) | -3.26%  (p=2.39x  10^-17^) | -4.28% (p=1.23x  10^-29^) | -2.68%  (p=0.003) | -3.70% (p=4.48x  10^-6^) | 1.06%  (p=1) |
